# RAGE pathway activation and function in chronic kidney disease and COVID-19

**DOI:** 10.3389/fmed.2022.970423

**Published:** 2022-08-09

**Authors:** Colleen S. Curran, Jeffrey B. Kopp

**Affiliations:** ^1^Critical Care Medicine Department, Clinical Center, National Institutes of Health, Bethesda, MD, United States; ^2^Kidney Disease Section, NIDDK (National Institute of Diabetes and Digestive and Kidney Diseases), National Institutes of Health, Bethesda, MD, United States

**Keywords:** receptor for advanced glycation end-products, macrophage antigen 1, S100A8, S100A9, High-mobility group box 1, complement C1q, lysophosphatidic acid, urokinase-type plasminogen activator receptor

## Abstract

The multi-ligand receptor for advanced glycation end-products (RAGE) and its ligands are contributing factors in autoimmunity, cancers, and infectious disease. RAGE activation is increased in chronic kidney disease (CKD) and coronavirus disease 2019 (COVID-19). CKD may increase the risk of COVID-19 severity and may also develop in the form of long COVID. RAGE is expressed in essentially all kidney cell types. Increased production of RAGE isoforms and RAGE ligands during CKD and COVID-19 promotes RAGE activity. The downstream effects include cellular dysfunction, tissue injury, fibrosis, and inflammation, which in turn contribute to a decline in kidney function, hypertension, thrombotic disorders, and cognitive impairment. In this review, we discuss the forms and mechanisms of RAGE and RAGE ligands in the kidney and COVID-19. Because various small molecules antagonize RAGE activity in animal models, targeting RAGE, its co-receptors, or its ligands may offer novel therapeutic approaches to slowing or halting progressive kidney disease, for which current therapies are often inadequate.

## Introduction

Chronic kidney disease (CKD) is caused by both systemic diseases (e.g., diabetes mellitus, systemic lupus erythematosus, systemic vasculitis, malignant hypertension and viral infections, including hepatitis B and HIV) and primary kidney diseases (e.g., podocytopathies, polycystic kidney disease, interstitial nephritis) (1). CKD also occurs in patients following infection with coronavirus disease 2019 (COVID-19). In meta-analyses of studies of hospitalized COVID-19 patients, acute kidney injury (AKI) occurred in ~30% of patients (2, 3) and in ~45–56% of patients of patients admitted to intensive care units (ICU), (3, 4). Patients with AKI and COVID-19 admitted to the hospital (5) or ICU (6) are also at substantial mortality risk. Moreover, patients who recover from COVID-19 may experience a post-COVID condition involving pulmonary, cardiac, neurologic, and renal disorders, termed long COVID. This includes an increased risk of AKI and end-stage kidney disease (ESKD) (7).

**Figure 1:**
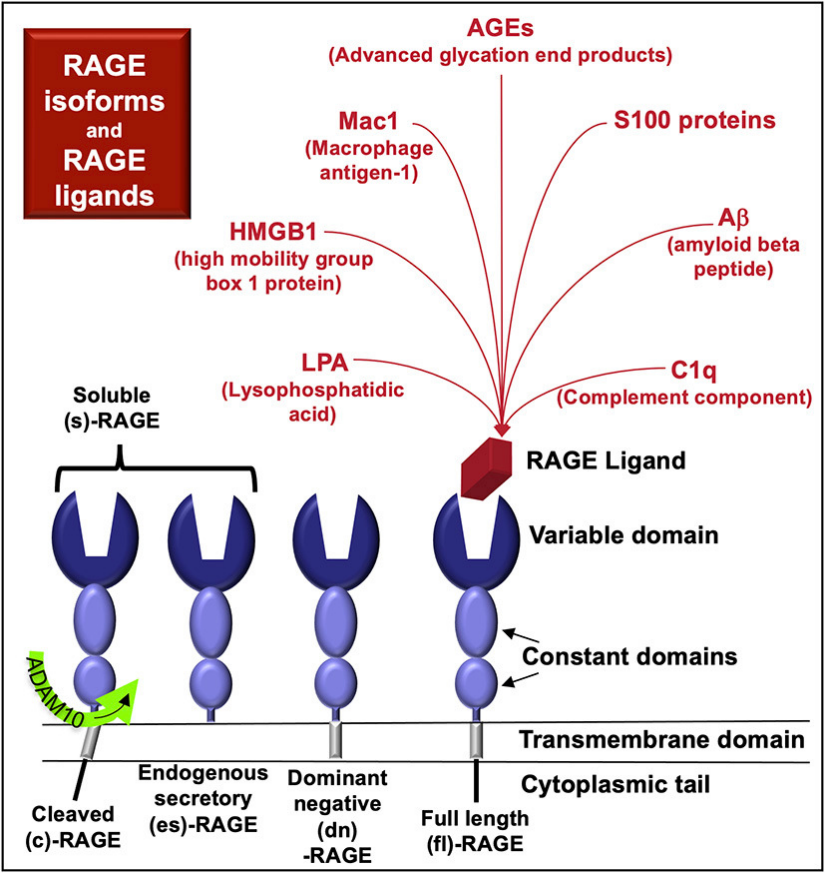
**RAGE and RAGE ligands**. RAGE variants characterized at the protein level include full-length (fl)-RAGE, endogenous secretory (es)-RAGE, and dominant negative (dn)-RAGE. RAGE may also be cleaved from the cell surface by proteases (cRAGE) and function similarly to esRAGE. RAGE in serum or plasma is termed soluble (s)-RAGE and can include cRAGE and/or esRAGE. Competitive binding interactions may exist among the various RAGE proteins for RAGE ligands.

**Figure 2:**
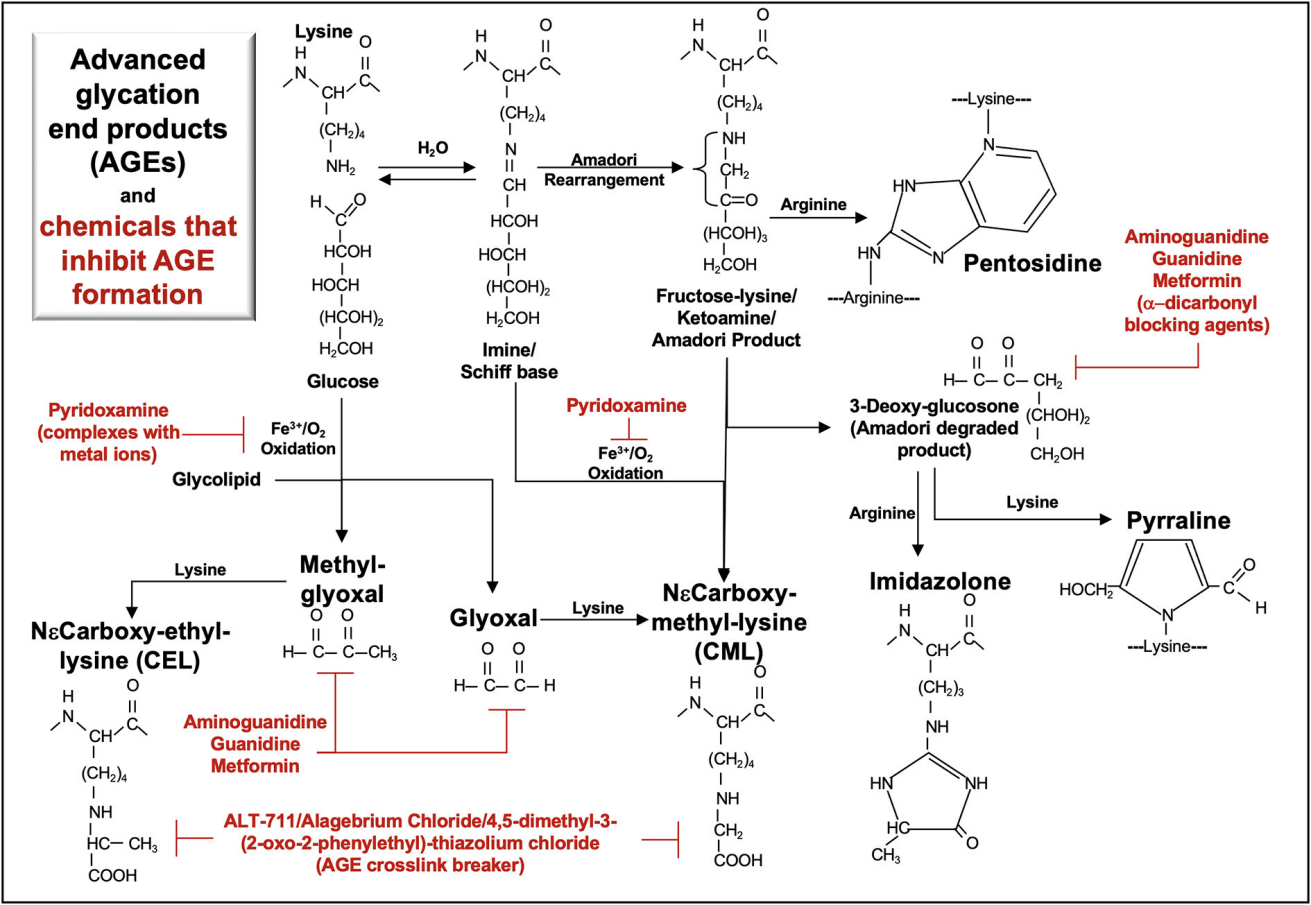
**Metabolic pathways of selected AGE and their inhibitors**. Advanced glycation end-products (AGE) are formed via non-enzymatic glycosylation of proteins. Glycation initially occurs when the aldehyde or ketone groups of reducing sugars react reversibly with an amino group as found in lysine, forming a Schiff base. Rearrangement of the Schiff base yields a more stable ketoamine (Amadori product), in which the free carbonyl may react with an arginine free amino group to form pentosidine. Oxidation of glycolipids, glucose, Schiff base or Amadori products yields highly reactive carbonyl intermediates (e.g., glyoxal, methyl-glyoxal, 3-deoxy-glucosone) that react with a free amino group, forming various AGEs (CEL, CML, pentosidine, pyrraline, imidazolone). Aminoguanidine, guanidine, metformin, pyridoxamine, and ALT-711 inhibit AGE formation.

The multi-ligand receptor for advanced glycation end-products (RAGE, Figure 1) is a biomarker of CKD and may also be a pathogenic factor in some kidney diseases. The primary RAGE ligands constitute a heterogeneous class of structures formed via non-enzymatic glycosylation, advanced glycation end-products (AGE, Figure 2) (8), that increase in circulation during CKD (9). RAGE is encoded by the gene *AGER*, located on human chromosome 6. The gene is within the major histocompatibility class III locus and near the genes for the transcriptional activator *PBX2* and cell surface receptor *neurogenic locus notch homolog protein-3* (*NOTCH3*) (10).

More than 20 mRNA splice variants of RAGE have been detected in human tissues (11). However, only four RAGE proteins have been characterized (Figure 1); it remains unclear whether the other RNA variants are translated and if so, in what tissues the proteins might be expressed.

There are four known naturally-occurring RAGE protein isoforms:

- full length (fl)-RAGE
- dominant negative (dn)-RAGE, which lacks a functional intracellular domain
- endogenous secretory (es)-RAGE, which lacks a transmembrane and intracellular domain
- cRAGE, generated by proteolytic cleavage from the cell-membrane, carried out by a disintegrin and metalloproteinase domain-containing protein 10 (ADAM10)

RAGE isoforms in serum and plasma are termed soluble RAGE (sRAGE) and include cleaved RAGE (cRAGE) and endogenous secretory RAGE (esRAGE) (11, 12) (Figure 1). RAGE plasma levels (sRAGE) are elevated in various diseases that cause CKD, including diabetes mellitus (13), hypertension (associated with arterionephrosclerosis) (14), systemic lupus erythematosus (15), and systemic vasculitis (16), as well as primary kidney diseases such as polycystic kidney disease (17).

RAGE isoforms form homo- and hetero-dimers that bind a diverse array of ligands (18). These ligands include small molecules, such as such as advanced glycosylation end-products (AGE, Figure 2) and lysophosphatidic acid (LPA) (19). Other RAGE ligands include plasma proteins released from monocytes/macrophages [e.g., complement component C1q (20, 21)] as well as intracellular transcriptional regulators [e.g., high-mobility group box-1 (HMGB1) (22)], cytoskeletal proteins [e.g., S100A8/S100A9, calprotectin (23)], and membrane molecules [e.g., amyloid-beta (24)] that bind RAGE upon their release from cells. RAGE ligands are also located on the cell surface and include macrophage antigen-1 (Mac-1), which is also known as CD11b/CD18; α_M_β_2_-integrin, and as complement receptor 3 (25).

RAGE and RAGE ligands are strongly implicated in severe acute respiratory syndrome coronavirus 2 (SARS-CoV-2) infections, which may initiate or exacerbate kidney injury. Acute and chronic inflammation in COVID-19 and CKD, respectively, drive pathogenesis, suggesting that RAGE activity, identified in both diseases, may illustrate the molecular mechanisms in which these syndromes interact. In order to explore the role of RAGE and its ligands in COVID-19-induced CKD, we review here the ligands and their interaction with RAGE in the kidney and with respect to SARS-CoV-2.

## RAGE expression in the kidney

RAGE is expressed in various cells in kidney, including podocytes, mesangial cells, tubular epithelial cells, neurons, endothelial cells, and infiltrating immune cells (26). In normal human kidney, both RAGE expression and AGE levels are low. By contrast, in diabetic nephropathy, there is increased AGE deposition and increased RAGE expression in most glomerular, tubulointerstitial, and vascular cells (27).

RAGE expression increases with age. In renal tissue from patients undergoing radical nephrectomy for urological tumors or trauma, younger patients (10 patients, <65 years old) expressed less RAGE and pSTAT5 (signal transducer and activator of transcription) compared to older patients (10 patients, >65 years old), as assessed by immunoblots (28). In C57BL/6N mouse kidneys, the binding affinities between RAGE and its ligands, e.g., AGE, S100B, HMGB1, also increases with age (comparing 3, 12, and 24 months). This increased binding is associated with increased tissue infiltration of activated macrophages (29).

In 142 CKD patients (average eGFR 32 ml/min/1.73 m^2^), plasma sRAGE is elevated compared to healthy controls and is inversely associated with estimated glomerular filtration rate, echocardiographic intima–media thickness, and the total number of atherosclerotic plaques (30). In a similar study of 142 CKD patients, elevated plasma sRAGE is inversely associated with echocardiographic left ventricular mass index and mean wall thickness (31). These studies suggest a role for sRAGE in preventing the development of atherosclerosis in CKD.

However, in 25 patients with ESKD (both hemodialysis and peritoneal dialysis) serum sRAGE levels are also elevated in comparison to 21 healthy subjects and 25 CKD patients (32). Because coronary artery disease occurs at a disproportionately high frequency in patients with ESKD (33), elevated sRAGE could also reflect vascular damage. The complexity of sRAGE is further highlighted in hemodialysis patients who exhibit a 50% increase in serum sRAGE from 0 to 15 min during hemodialysis or hemodiafiltration (32). The source of sRAGE in response to hemodynamic sheer stress could be endothelial cells or additional cells in the vasculature and/or kidney microenvironment. Whether the increase in sRAGE in CKD and ESKD patients is limited to inhibiting cell surface RAGE activation during disease progression is not clearly known. To further understand RAGE function, we have reviewed the current literature on RAGE in the various cell types in the kidney. The downstream consequences of RAGE homodimers and complexes in response to RAGE ligands in podocytes, mesangial cells, epithelial cells, and endothelial cells are discussed and displayed in the associated figures below.

## Podocytes

In the *db/db* mouse model of type 2 diabetes, RAGE expression is elevated in podocytes and there is increased recruitment of S100A8/S100A9 (calprotectin)-expressing leukocytes into glomeruli. Therapeutic administration of sRAGE to these mice reduces inflammation and improves renal function (34).

AGE binding to RAGE-expressing podocytes induces γ-secretase activity, which increases cleavage of the signaling protein NOTCH1. As shown in studies of AGE-exposed human podocytes and in a mouse model involving intra-peritoneal injections of *in vitro*-prepared AGE, NOTCH1 cleavage generates the transcriptional co-activator, NOTCH1 intracellular domain (NICD1) (35). This in turn activates pathways involving the expression of the transcription factor, Hairy and Enhancer of Split (HES1), and production of the NOTCH ligand, Jagged-1 (JAG1). NOTCH activation in podocytes induces injury (36), suggesting that RAGE-induced γ-secretase activity may contribute to podocyte injury.

Further, mouse metanephroi cultured in the presence of a γ-secretase inhibitor, N-S-phenyl-glycine-t-butyl ester (DAPT), manifest impaired NOTCH1 signaling and podocyte development (37). Increased NOTCH1 signaling is present in mouse podocytes exposed to angiotensin (Ang)-II (38). Further data from cultured podocytes suggests that this may occur through increased RAGE production via Ang II-induced activation of the angiotensin II type 2 receptor (AT2R) (39) and subsequent RAGE ligand activation of γ-secretase (35). Taken together, these studies indicate a role for RAGE and its ligands in podocyte development and podocyte function.

The angiotensin II type 1 receptor (AT1R) forms a heteromeric complex with RAGE, and Ang II-induced activation of the AT1R transactivates RAGE (40). Both AT1R and RAGE activate a downstream signal, diaphanous 1 (mDia1/DIAPH1), which is an actin-associated protein involved in the regulation of cell morphology and cytoskeletal organization (40, 41). Deletion of *Diaph1* in streptozotocin-induced diabetic C57BL/6 mice suppresses glomerular inflammation and reduces podocyte foot-process effacement (41). Expression of DIAPH1 in human podocytes and Diaph1 in murine podocytes (41) is required for RAGE cell signaling (18). A downstream signal of RAGE/DIAPH1 in mouse peritoneal macrophages is Rac1, which is inhibited by an antagonist to cRAGE/DIAPH1 (42). In hyperhomocysteinemia mice, intraperitoneal administration of a Rac1 activator (UTP) promoted whereas a Rac1 inhibitor (NSC-23766) blocked inflammasome activation, podocyte injury, and glomerulosclerosis (43). Possibly, a cRAGE/DIAPH1 inhibitor could also have a similar effect in CKD.

RAGE also co-immunoprecipitates with the αVβ3-integrin (44), which is activated by a glycosylated cleaved product of the cell-bound urokinase-type plasminogen activator receptor (uPAR). This multifunctional receptor binds urokinase plasminogen activator (uPA), which cleaves plasminogen to plasmin and promotes fibrinolysis, matrix remodeling, and migration. Cleavage of the uPAR GPI anchor by phospholipases releases uPAR as a soluble form (suPAR), which can bind αVβ3-integrin and the chemotactic receptor lipoxin A4 receptor (LXA4R/FPR1) (45).

Studies of suPAR in murine MPC-5 podocytes indicate that the ligand increases the production of reactive oxygen species (ROS) through the NADPH oxidase 2 (NOX2) in a Rac-1-dependent manner. This oxidative stress then promotes the activation of Src kinases and the increased production of TRPC6 calcium channels (46), which are additionally important to podocyte cytoskeletal organization, adhesion, and motility (47). In examining a function of RAGE in MPC-5 podocytes, AGE also induced Src kinase and Rac1 activation and production of ROS and TRPC6. Blocking with a RAGE antagonist (FPS-ZM1, azeliragon) or an αVβ3 integrin antagonist (cilengitide) inhibited the downstream responses. Moreover, RAGE knockdown also attenuated Src phosphorylation induced by suPAR or AGE in cultured podocytes, further suggesting that RAGE functions as a co-receptor with αvβ3-integrin and as a possible therapeutic target in regulating suPAR cell signals (44).

**Figure 3:**
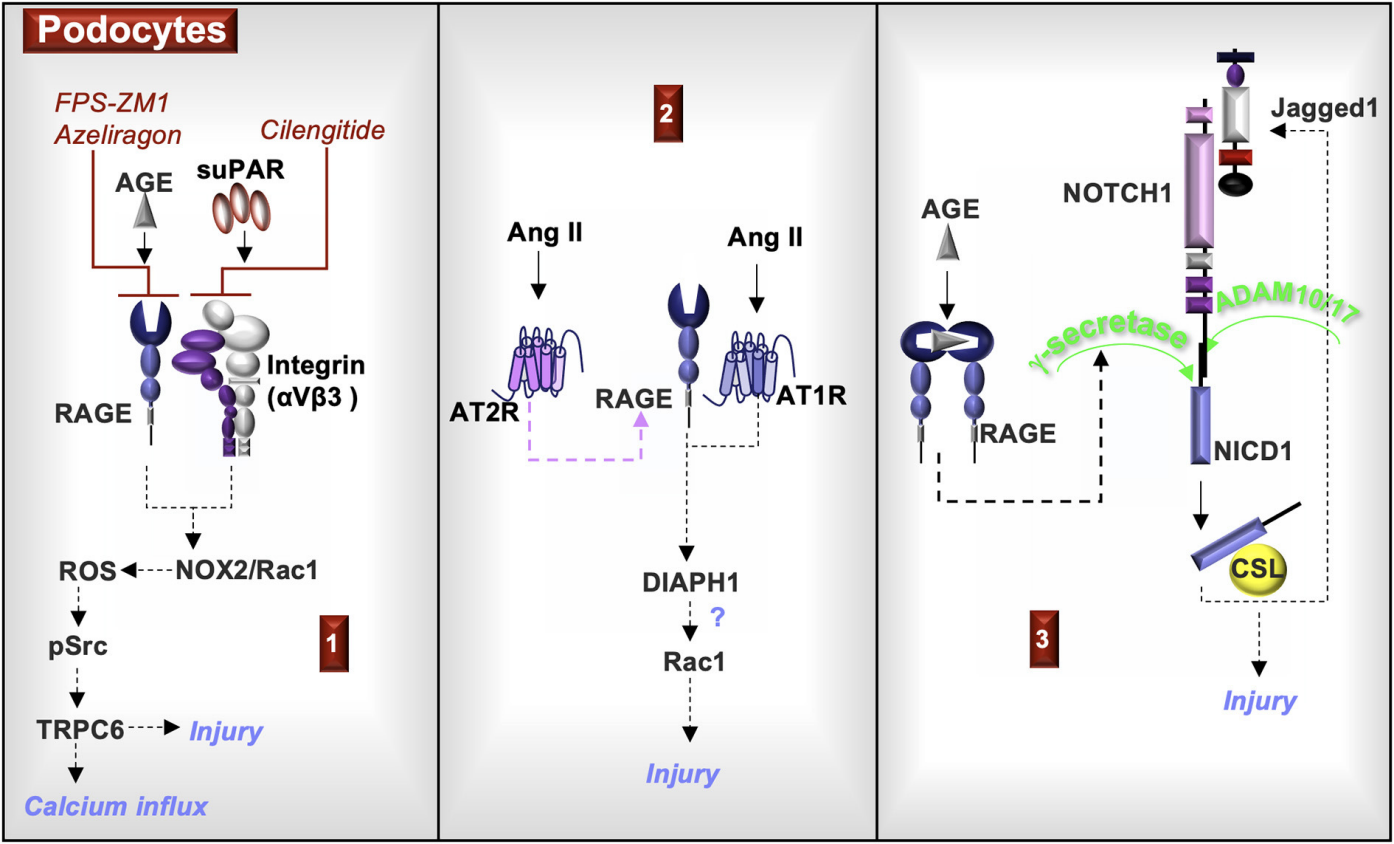
**RAGE signaling in podocytes**. **(1)** Exposure of podocytes to either AGE or soluble urokinase plasminogen activator receptor (suPAR) activates the NADPH oxidase 2 (NOX2) and Rac1, which is a component of the NOX2 multiprotein complex. NOX2 generates reactive oxygen species (ROS) that activates Src kinases and induces the production of the calcium channel, TRPC6. Each of these responses can be inhibited by either RAGE antagonists (FPS-ZM1or azeliragon) or by an αVβ3 integrin antagonist (cilengitide, an angiogenesis inhibitor). Changes in TRPC6 activity are associated with podocyte injury. **(2)** Angiotensin II (Ang II) activation of the Ang II type 2 receptor (AT2R) induces RAGE expression. Ang II also activates AT1R, which transactivates RAGE and induces the common RAGE downstream signal, diaphanous homolog 1 (DIAPH1). In additional cell types, DIAPH1 induces Rac1 activity, suggesting a potential similar function in podocytes. Rac1 activity can damage podocytes and induce foot process effacement. **(3)** AGE activation of RAGE induces the production of γ-secretase, which cleaves NOTCH1 to form the transcription co-activator, NOTCH1 intracellular domain (NICD1). NICD1 complexed to CBF-1/suppressor of hairless/LAG-1 (CSL) promotes the transcription of the NOTCH ligand, Jagged1. NOTCH1 cell signals in podocytes are associated with injury.

In summary, RAGE is expressed in podocytes, which may encourage the recruitment of S100A8/S100A9-positive leukocytes. Activation of RAGE in podocytes induces γ-secretase and NOTCH activity associated with injury. Ang II promotes RAGE expression through AT2R and induces RAGE transactivation via AT1R. A downstream signal of RAGE and AT1R is DIAPH1, which can induce the activation of Rac1 in macrophages and possibly other cell types, such as podocytes. In podocytes, activation of Rac1 promotes injury. RAGE and αVβ3 complex and regulate the activity of TRPC6, which is associated with calcium transport, adhesion, motility, and podocyte homeostasis. Changes in TRPC6 function are associated with podocyte injury (Figure 3).

## Mesangial cells

In normal rat glomerular mesangial cells, AGE induce Smad2 and Smad3 phosphorylation, which in turn promote profibrotic type I collagen production (27). In primary rat mesangial cells, AGE induce intracellular ROS, Ang II, and transforming growth factor (TGF)-β1 production; all of these mediators are antagonized by exposure to the AT1R blocker, candesartan (48). This observation suggests a cell signal cascade involving RAGE-induced ROS. Higher ROS levels increase the activity of angiotensin-converting enzyme (ACE), which cleaves Ang I to Ang II, leading to Ang II-induced TGF-β1 production via AT1R cell signaling (1, 48). Thus, AT1R and RAGE signaling may contribute to mesangial cell type I collagen production identified in human sclerotic glomerular lesions (49).

In the murine mesangial cell line SV40 MES 13, both glyoxal-lysine dimers and methyl glyoxal-lysine dimers (AGE) increase production of RAGE and ROS (50, 51). The downstream effects of RAGE-induced ROS in rat mesangial cells include reduced levels of the antioxidant transcription factor nuclear factor erythroid 2-related factor 2 (NRF2) (50) and increased activation of phosphoinositide 3-kinase (PI3K)/AKT pathway, which leads to activation of nuclear factor (NF)-κB and the production of pro-inflammatory cytokines (51).

**Figure 4:**
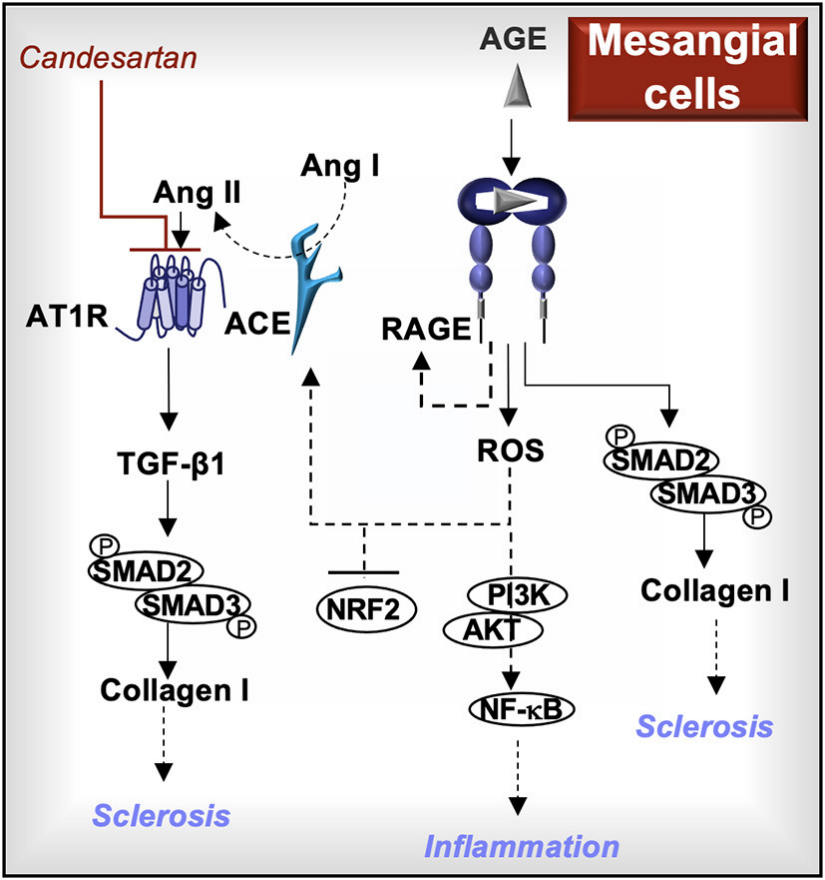
**RAGE signaling in mesangial cells**. Activation of RAGE in mesangial cells further increases RAGE production and induces the generation of reactive oxygen species (ROS). The downstream effects of RAGE-induced ROS may include enhanced PI3K/AKT/NF-kB cell signaling, reduced production of the antioxidant transcription factor, nuclear factor erythroid 2-related factor 2 (NRF2), and increased angiotensin-converting enzyme (ACE) activity. ACE cleaves angiotensin I (Ang I) into Ang II, which activates the Ang II type I receptor (AT1R) and induces the production of transforming growth factor (TGF)-β1. Both TGF-β1 and RAGE induce the phosphorylation of SMAD2 and SMAD3, which are involved in the production of type I collagen. AT1R blockers (e.g., candesartan) and/or RAGE inhibitors may reduce the development of sclerotic lesions and inflammation associated with glomerulosclerosis.

In summary, mesangial RAGE promotes the activity of SMAD2/3 and production of type I collagen, similar to TGF-β1. RAGE-induced ROS contribute to ACE/Ang II/AT1R pathway activation, which also activates SMAD2/3 indirectly through TGF-β1 production. Mesangial AT1R and RAGE activation cooperate in promoting inflammation and fibrosis in glomerulosclerosis, indicating that inhibitors to AT1R and/or RAGE may impede disease progression (Figure 4).

## Epithelial cells

Similar to their effects on mesangial cells, AGE also induce the production of RAGE, ROS, TGF-β1 and type I collagen in tubular epithelial cells *in vitro* (27, 52). The dimethylbiguanide metformin inhibits the formation of α-dicarbonyl AGE (53) (Figure 2) and also activates AMP-activated protein kinase (AMPK)-α (54), improving mitochondrial function (55). In human proximal tubule epithelial cells, metformin blocks AGE-induced RAGE expression and ROS production and this response is abrogated by an AMPK-α inhibitor (C compound) (52).

Although the mechanisms involved are not fully elucidated, AMPK-α regulation of RAGE could involve increased tubular epithelial cell production of the antioxidant thioredoxin (56). AMPK-α may also regulate RAGE-induced NF-κB (57) by increasing intracellular NAD levels, which promote SIRT1 activity and the deacetylation of target molecules (58). In a murine cisplatin-induced AKI model, administration of the peroxisome proliferator-activated receptor γ (PPAR-γ) agonist, pioglitazone, protected the kidney from cisplatin injury by promoting AMPK phosphorylation, SIRT1 activation and deacetylation/inactivation of the NF-κB subunit p65 (59). Because NF-κB also binds to and activates the RAGE promoter (60), the functions of AMPK-α in regulating RAGE merit further study.

AGE-induced type I collagen production in epithelial cells is regulated by both mitogen-activated protein kinase (MAPK)-induced/TGF-β1-independent and TGF-β1-dependent Smad cell signals (27). AGE-induced transdifferentiation of rat kidney epithelial cells (NRK-52E) into myofibroblasts is also dependent upon both TGF-β1 (61) and MAPK (62) cell signals, possibly through downstream Smad3-induced α-smooth muscle actin (SMA) expression (63).

Moreover, both AGE-induced MAPK activity and epithelial mesenchymal transition (EMT) in rat NRK-52E cells are blocked by treatment with AT1R blockers (e.g., olemesartan, irbesartan, losartan) (64). In NRK-52E epithelial cells, DIAPH1 is a necessary factor in the production of TGF-β1, type I collagen, profilin1, and α-SMA. Absence of DIAPH1 in TGF-β1 stimulated NRK-52E cells reduces the production of these same factors, which promote EMT and migration (65). DIAPH1 associates with the RAGE cytoplasmic tail in initiating cell signal transduction (66) and in murine tubule epithelial cells, AGE activate DIAPH1 (41). Whether DIAPH1 downstream of RAGE functions similarly to DIAPH1 downstream of TGF-β1 requires further study. Research in human renal tubular epithelial HK-2 cells indicates that production of EMT-associated factors (e.g., TGF-β1, α-SMA) are dependent on RAGE-induced release of HMGB1(67), highlighting the strong interdependency of RAGE and TGF-β1 cell signals.

**Figure 5:**
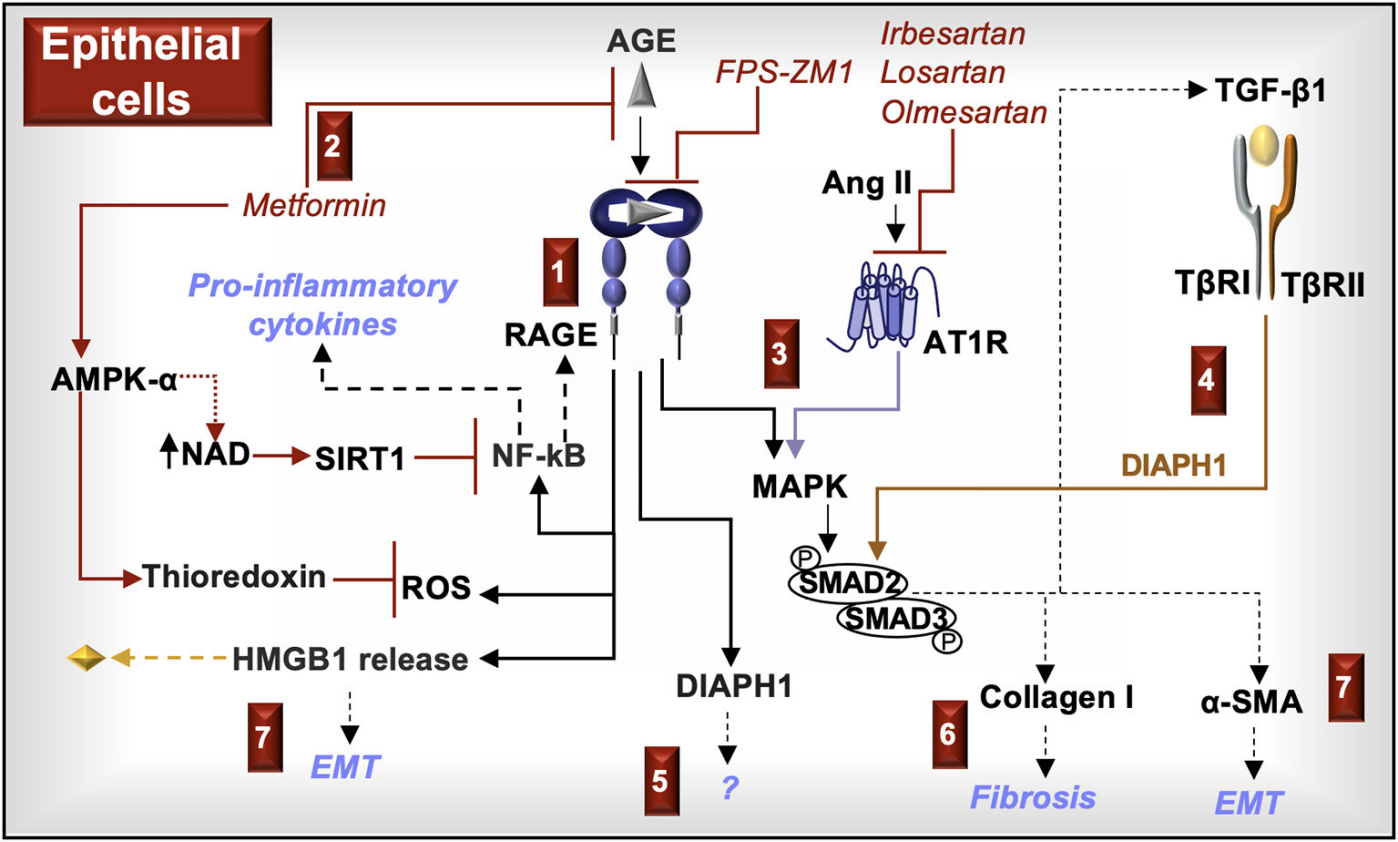
**RAGE signaling in epithelial cells**. **(1)** AGE activation of RAGE in kidney epithelial cells increases reactive oxygen species (ROS), induces the activation of NF-κB, and promotes the release of HMGB1. ROS in association with NF-κB-induced cytokines and increased RAGE expression promote inflammation. **(2)** Metformin, in addition to being a hypoglycemic agent, prevents RAGE signaling by inhibiting the formation of α-dicarbonyl AGE and activating AMP-activated protein kinase (AMPK)-α, which increases the production of NAD and the antioxidant, thioredoxin. NAD promotes the activity of sirtuin-1 (SIRT1) and deacetylates/inactivates the NF-κB subunit p65. **(3)** RAGE and the Ang II type I receptor (AT1R) activate MAPK (e.g., ERK/p38 MAPK) and SMAD2/3, which promote the production of TGF-β1, type I collagen, and α-smooth muscle actin (SMA). **(4)** TGF-β1 induces SMAD2/3 and their downstream responses in a DIAPH1-dependent manner. **(5)** DIAPH1 is directly activated by the cytoplasmic tail of RAGE. RAGE-induced DIAPH1 functions in kidney epithelial cells are not fully known. **(6)** Type I collagen deposition promotes fibrosis. **(7)** HMGB1 promotes the production of TGF-β1 and α-SMA in a RAGE dependent manner. Increased production of α-SMA promotes epithelial mesenchymal transition (EMT).

In summary, RAGE activation in epithelial cells induces cell signals (DIAPH1, MAPK, SMAD2/3, NF-κB) and promotes the production of molecules (ROS, TGF-β1, type I collagen, α-SMA, HMGB1) commonly associated with fibrogenesis, EMT, and inflammation. Because RAGE complexes with AT1R in these cells, AT1R blockers antagonize RAGE cell signals. Metformin inhibits the formation of α-dicarbonyl AGE and may antagonize RAGE-induced ROS production and NF-κB activity through activation of AMPK-α. Molecules that block AT1R and RAGE and/or promote AMPK-α activation may attenuate the fibrotic and inflammatory responses of RAGE in tubular epithelial cells (Figure 5).

## Leukocytes and endothelial cells

Recruitment of monocytes and macrophages into the kidney is common in many forms of CKD (68), whereas the presence of neutrophils suggests bacterial infection, vasculitis, and occasionally other disorders. Neutrophils contain a Ca^2+^ and Zn^2+^ binding dimer (S100A8/S100A9, also known as calprotectin), estimated at more than 40% and 5% of cytosolic and total proteins of neutrophils, respectively. These proteins bind tubulin to regulate cytoskeleton organization, phagocytosis, and migration and are abundantly released in various non-infectious and infectious diseases to promote chemotaxis, cell signaling, and apoptosis (69). In patients with active antineutrophil cytoplasm antibody (ANCA)–associated vasculitis (AAV), renal biopsies demonstrate glomerular infiltration of S100A8/S100A9-positive immune. The patients also exhibited increased circulating S100A8/S100A9-positive monocytes and neutrophils, and increased serum S100A8/S100A9 levels (70). In a murine model of nephrotoxic nephritis, S100A8/S100A9-positive macrophages are recruited into glomeruli and serum levels of S100A8/S100A9 are elevated (71).

Leukocyte adhesive interactions with the endothelium may involve S100A9-induced upregulation of Mac-1 on leukocytes (72), which binds RAGE and/or intercellular adhesion molecule (ICAM)-1 on the endothelial cells (73). S100A8/S100A9 activation of RAGE or Ang II activation of AT1R induce ICAM-1 expression in primary murine aortic endothelial cells, which can be blocked with siRNA specific to RAGE or to the NF-κB–p65 subunit (40), further supporting a role for RAGE and its ligands in leukocyte recruitment to the endothelium.

Moreover, co-cultures of renal microvascular endothelial cells from wild-type mice and bone marrow–derived macrophages (BMDMs) derived from either wild-type or S100A9^−/−^ mice results in increased cytokine production in co-cultures of endothelial cells and wildtype BMDMs compared to endothelial cell and S100A9^−/−^ BMDM co-cultures or cultures of BMDMs or endothelial cells alone. Specifically, wild-type co-cultures generated increased levels of the mediators C-X-C motif chemokine ligand 1 (CXCL1), interleukin (IL)-6, and C-C motif chemokine ligand 2 (CCL2), the latter accumulating to the highest levels (71). Pre-incubating human umbilical vein endothelial cells (HUVEC) with AGE prior to exposure to S100A8/S1009 heterodimers increases the production of IL-6 and CCL2 in a dose-dependent manner (74), indicating that CKD patients with AGE-generating co-morbidities may be at risk for enhanced vascular inflammation.

**Figure 6:**
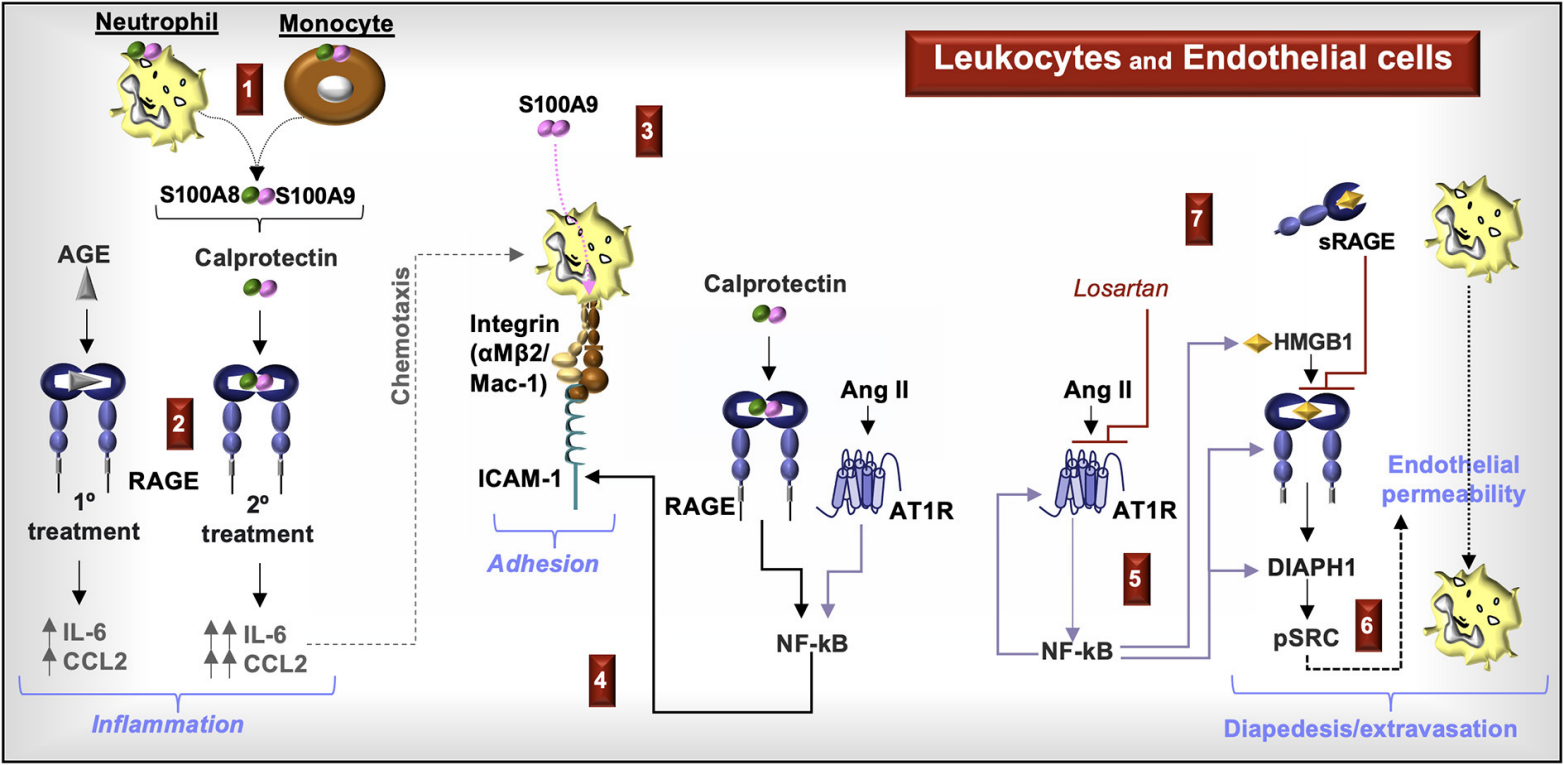
**RAGE signaling in endothelial cells and leukocytes**. **(1)** Increased circulating calprotectin-positive monocytes and neutrophils and increased serum calprotectin levels are characteristic of CKD. **(2)** Priming endothelial cells with AGE prior to stimulation with calprotectin increases the production of chemokines CCL2 and IL-6. **(3)** S100A9 induces leukocyte cell surface expression of Mac-1. **(4)** Calprotectin activation of RAGE and Ang II activation of the Ang II type I receptor (AT1R) induces NF-kB and increased cell surface expression of intracellular adhesion molecule (ICAM)-1, which enhances leukocyte adhesion to Mac-1. **(5)** Ang II-induced NF-kB promotes the expression and production of AT1R, RAGE, DIAPH1, and HMGB1. **(6)** Ang II induces endothelial permeability by inducing the phosphorylation of Src in a RAGE- and DIAPH1-dependent manner. **(7)** Blocking AT1R with an AT1R blocker, losartan, or RAGE with soluble RAGE (sRAGE) reduces endothelial permeability, which facilitates leukocyte diapedesis and extravasation.

Lastly, Ang II-induced NF-κB in HUVECs increases production of AT1R and promotes autocrine RAGE activation by upregulating HMGB1 and RAGE. Ang II-induces DIAPH1 expression and downstream pSRC-induced endothelial layer permeability in cultured HUVECs through RAGE activation. Blocking RAGE with sRAGE or blocking the AT1R with a specific antagonist (e.g., losartan) reduces endothelial permeability (75) (Figure 6).

In summary, S100A8/S100A9 molecules and S100A8/S100A9-positive myeloid cells increase in circulation during CKD. RAGE ligands induce endothelial chemokine production associated with the recruitment of immune cells, upregulate myeloid Mac-1 cell surface expression, and increase endothelial cell surface ICAM-1 expression. Ang II supports leukocyte adhesion to the endothelium through NF-κB activation and enhances RAGE signaling. Activation of Src kinases, downstream of RAGE-associated DIAPH1, promotes endothelial permeability. Inhibitors to RAGE, AT1R, and DIAPH1 may therefore protect the endothelium during CKD.

## RAGE, the kidney and COVID-19

As described above, RAGE is implicated in CKD pathogenesis through the expression and function of the protein in various cells of the kidney and immune system. Small molecules and biologics that target RAGE, RAGE ligands, and RAGE downstream cell signals in models of CKD repeatedly have demonstrated reduced kidney pathology and improved function (Table 1). Crosstalk between RAGE and various receptors expands the complexity of RAGE cell signals and responses to molecular RAGE inhibition. Specifically, the interactions between RAGE and NOTCH (Figure 3) or AT1R (Figures 3–6) are unique to kidney function and possibly to COVID-19.

**Table 1:** Possible approaches to inhibiting RAGE in treating CKD.

**Source**	**Treatment**	**Model**	**Experimental Results**
Mouse	RAGE inhibitor FPS-ZM1 or the γ-secretase inhibitor DAPT	C57BL/6J mice were administered AGE (10 mg/kg) +/– DAPT (10 mg/kg), or FPS-ZM1 (1 mg/kg), daily, for 4 weeks	Inhibitors of RAGE or γ-secretase activity ameliorated AGE-induced kidney glomerular fibrosis, thickening of the glomerular basement membrane, foot process effacement, and proteinuria (35)
Mouse	RAGE inhibitor FPS-ZM1	Male CD1 mice administered intraperitoneal AGE-BSA	FPS-ZM1 attenuated urinary albumin levels in AGE-loaded CD1 diabetic mice (76)
Mouse	A small-molecule antagonist of cRAGE-DIAPH1 interaction, termed RAGE229	C57BL6 mice with streptozotocin-induced diabetes at 8 weeks were subsequently fed RAGE299-containing chow or control chow for 6 months	Treatment with 150 or 50 ppm/day RAGE299 in chow attenuated mesangial sclerosis, tubular atrophy, podocyte effacement, and interstitial fibrosis (77)
Mouse	Recombinant adeno-associated virus-mediated expression of esRAGE *in vivo*	BALB/c mice with streptozotocin-induced diabetes	Mice expressing esRAGE exhibited reduced glomerular injury and interstitial fibrosis, which may be a result of HMGB1 neutralization (78)
Mouse	AGE crosslink breaker, alagebrium/ALT-711 or the ACE inhibitor, quinapril	Male *apoE* KO and *RAGE apoE* double-KO C57BL6 mice with streptozotocin-induced diabetes	RAGE deletion attenuated mesangial expansion and reduced glomerular collagen IV deposition. Both alagebrium and quinapril reduced AGE formation, glomerular fibrosis, and attenuated renal inflammation in diabetic *RAGE apoE* double-KO mice. Only quinapril lowered albumin excretion, highlighting a RAGE-independent ACE inhibitor-mediated response (79)
Mouse	Ursolic acid or metformin	Swiss albino mice with alloxan-induced diabetes were orally administered metformin (1, 50, or 100 mg/kg) or ursolic acid (1, 50, or 100 mg/kg)	Ursolic acid and metformin inhibited AGE formation in serum and the kidney and improved blood lipid profiles, kidney function, and liver enzymes in a dose-dependent manner (80)
Mouse	Antioxidant, vitamin B6 analog, and inhibitor of AGE formation, pyridoxamine	BALB/c mice ischemia-reperfusion-induced AKI mice given 2.5 and 5 g/mL pyridoxamine in drinking water, 72 h before or 24 h after injury	Pre-injury treatment with pyridoxamine reduced acute tubular injury, fibrosis, and oxidative stress in a dose-dependent manner. Renal function improved late (28 days) but not early (9 days) after injury as measured by serum creatine. Post-injury treatment reduced renal fibrosis, but renal function did not improve at early or late time points (81)
Mouse	Antioxidant, vitamin B6 analog, and inhibitor of AGE formation, pyridoxamine	C57Bl/6j mice fed a high-fat high-fructose diet supplemented with 1 g/L pyridoxamine in drinking water, over 12 weeks	Mice developed kidney vacuolar degeneration, a complete loss of the brush border integrity, fibrosis, and decreased renal function as measured by serum creatine, which were each ameliorated by pyridoxamine. Levels of AGE in tissue and hyperexpression of RAGE was also reduced by pyridoxamine (82)
Rat	AGE crosslink breaker, Alagebrium/ALT-711	Streptozotocin-induced diabetic Sprague Dawley rats were gavaged with ALT-711 (10 mg/kg/ day)	Rats that received alagebrium/ALT-711 exhibited reduced immunohistochemical staining for AGE α-SMA, and TGF-β in tubules (61)
Rat	Berberine, an isoquinoline alkaloid derived from plants	Streptozotocin-induced diabetic Sprague-Dawley rats administered intragastric berberine, 50–200 mg/kg, daily	Berberine improved kidney function and reduced mesangial expansion, glomerular hypertrophy, and recruitment of immune cells to the tubulointerstitium, comparable to intragastric administration of 200 mg/kg metformin and 15 mg/kg captopril, daily. Berberine also reduced the identification of AGE, RAGE, and TGF-β in the kidney (83)
Rat	RAGE inhibitor FPS-ZM1 and an angiotensin II receptor blocker	Tubular injury in streptozotocin-induced diabetic rats	FPS-ZM1 and valsartan reduced inflammation, oxidative stress and fibrosis and improved kidney function more significantly than either inhibitor alone (84)
Rat	Antioxidant, vitamin B6 analog, and inhibitor of AGE formation, pyridoxamine	Zucker (fa/fa) obese and lean (Fa/fa) rats were treated with 2 g/L pyridoxamine in drinking water, over 32 weeks	In the obese mice, dyslipidemia, decreased renal function, increased systolic blood pressure, increased thickening of the aortic wall, and increased AGE in skin were each ameliorated by pyridoxamine to levels comparable to the controls
Rat	A secoiridoid glycoside derived from plants, termed swertiamarin (SM) or aminoguanidine or metformin	Sprague Dawley rats were fed standard chow or a high fat diet (HFD) +/– intraperitoneal aminoguanidine (100 mg/kg), oral metformin (70 mg/kg), or oral SM (50 mg/kg)	SM was a more potent inhibitor of kidney fibrotic marker mRNA (e.g., TGF-β, collagen IV, fibronectin) and protein (e.g., TGF-β, heme oxygenase-1) than aminoguanidine or metformin compared to HFD alone. SM also improved kidney histopathology and reduced the levels of AGE in serum and kidney tissue (85)
Rat	α-dicarbonyl inhibitor aminoguanidine	Streptozotocin-induced diabetic Sprague Dawley rats were given drinking water with 2 g/L aminoguanidine for 3 weeks	Iodinated-AGE bound at a higher intensity to diabetic kidney renal tubule tissue compared to controls and the intensity was reduced in rats given aminoguanidine (86)
Rat	α-dicarbonyl inhibitor aminoguanidine	Streptozotocin-induced diabetic Sprague Dawley rats were given drinking water with 1 g/L aminoguanidine for 4 weeks	Increased AGE deposition in both the tubulointerstitium and glomerulus, increased renal expression of TGF-β1, PDGF-B mRNA, and type IV collagen accumulation were all attenuated in rats given aminoguanidine (87)
Rat	Licorice plant extract that binds and neutralizes HMGB1, glycyrrhizin	Streptozotocin-induced diabetic Sprague Dawley rats were given glycyrrhizin intragastrically (150 mg/kg/day) for 8 weeks	Diabetic mice recruited more CD14+ cells to renal tissue, produced more pro-inflammatory cytokines (IL-6, IL-1β) in serum, expressed higher mRNA levels of ICAM-1 and TGF-β1, and generated more RAGE, HMGB1, and TLR-4 protein compared to controls and rats given glycyrrhizin (88)
Rat	Recombinant sRAGE	Sprague Dawley rats were administered intraperitoneal sRAGE (150 μg/per rat) every 48 h for 5 weeks before being challenged with chronic intermittent hypoxia over 5 weeks	In rats exposed to chronic intermittent hypoxia, prior intraperitoneal injections of recombinant sRAGE reduced tubular atrophy, inflammatory cell infiltration, kidney function, endothelial apoptosis and kidney tissue p38 activation (89)

AGE-exposed human podocytes *in vitro* induce NOTCH1 cleavage and formation of NICD1 through the production of γ-secretase (35). RAGE activation also promotes Th1 immunity (90). In a preprint involving children with SARS-CoV-2-associated multisystem inflammatory syndrome, T-regulatory cells express increased cell surface NOTCH1, which reduces their suppressive functions (91). The role of RAGE in this response remains to be defined.

The angiotensin-converting enzyme (ACE)-2 is also regulated by γ-secretase. After ACE2 cell surface cleavage by ADAM10 or TMPRSS2, the γ-secretase enzyme forms an ACE2 intracellular domain, which like NICD1, may serve as a cell signaling molecule (92). ACE2 is a SARS-CoV-2 receptor and also an enzyme that counteracts angiotensin-converting enzyme (ACE) activity in the renin-angiotensin-aldosterone system (RAAS) (93). In the RAAS system, ACE promotes the cleavage of angiotensin I (Ang I) into Ang II, which binds to AT1R, thus promoting vasoconstrictive, thrombotic, inflammatory, and fibrotic effects. ACE2 cleaves Ang I and Ang II into peptides that inhibit ACE/Ang II/AT1R signaling and promote activation of additional receptors (e.g., Mas, MrgD, or AT2R) in maintaining a balance between inflammation/tissue injury and restoration of tissue integrity/function (93).

In humans and experimental animals, ACE2 is expressed in renal tubular cells, podocytes, mesangial cells, and endothelial cells. In renal biopsies from patients with type 2 diabetes, immunohistochemical expression of ACE is higher but ACE2 is lower in the glomeruli and tubulointerstitium compared to non-diabetic control biopsies (94). In diabetic animals, renoprotection is reduced in the absence of ACE2 (95). In a meta-analysis of patients with non-dialysis CKD (stages 3–5), ACE inhibitors were deemed superior to AT1R blockers (ARBs) in lowering probability of kidney events (e.g., doubling of serum creatinine level, 50% decline in GFR, or ESKD) but in a subgroup of patients with diabetic kidney disease, ARBs were superior to ACE inhibitors (96). Thus, promoting the activity and downstream signaling from ACE2 and antagonizing ACE are important in reducing CKD progression.

Because SARS-CoV-2 binding to ACE2 induces ACE2 degradation (92), the renoprotective functions of ACE2 and its enzymatic products are reduced, thus increasing the pathological activity of the ACE/Ang II/AT1R pathway. This may include Ang II-induced activation of neutrophils (97), polarization of macrophages to an M1 (pro-inflammatory) phenotype (98), and transactivation of RAGE in kidney parenchymal cells (40).

In human-*ACE2* transgenic mice infected with SARS-CoV-2, treatment with the RAGE inhibitor, FPS-ZM1, reduced overall inflammation and significantly increased survival (99). Thus, SARS-CoV-2-induced RAAS dysregulation may promote kidney injury and inflammation through RAGE.

## Soluble RAGE and COVID-19

COVID-19 patients manifest increased levels of sRAGE in plasma (100) and serum (101). These increases might arise via various processes: (1) pre-existing conditions (e.g., diabetes, cardiovascular disease, obesity), (2) as a response to increased epithelial/endothelial damage, and/or (3) as a result of SARS-CoV-2 activation of proteases which occurs subsequent to SARS-CoV-2 binding to and modulating the activity of ACE2 (102). The production of serum sRAGE in asymptomatic patients (≥10 ng/mL) with lower numeric age (average age 43 y) compared to sRAGE levels (<10 ng/mL) in symptomatic elderly patients (average age 62 y) (103) may suggest a protective role of sRAGE.

However, in a study involving 164 COVID-19 patients, a serum sRAGE ≥3.1 ng/mL predicted the need for mechanical ventilation and a value ≥5.8 ng/mL predicted higher 30-day mortality (101). In a retrospective study of 33 COVID-19 patients, 11 with and 22 without diabetes, serum sRAGE levels were elevated in both groups and the levels of the cleaved form were higher compared to esRAGE in patients, regardless of diabetes mellitus status (104). Whether differences in the composition of sRAGE (e.g., cRAGE vs. esRAGE, Figure 1) affect ligand affinity for membrane-bound flRAGE requires further study.

## RAGE ligands and COVID-19

**Figure 7:**
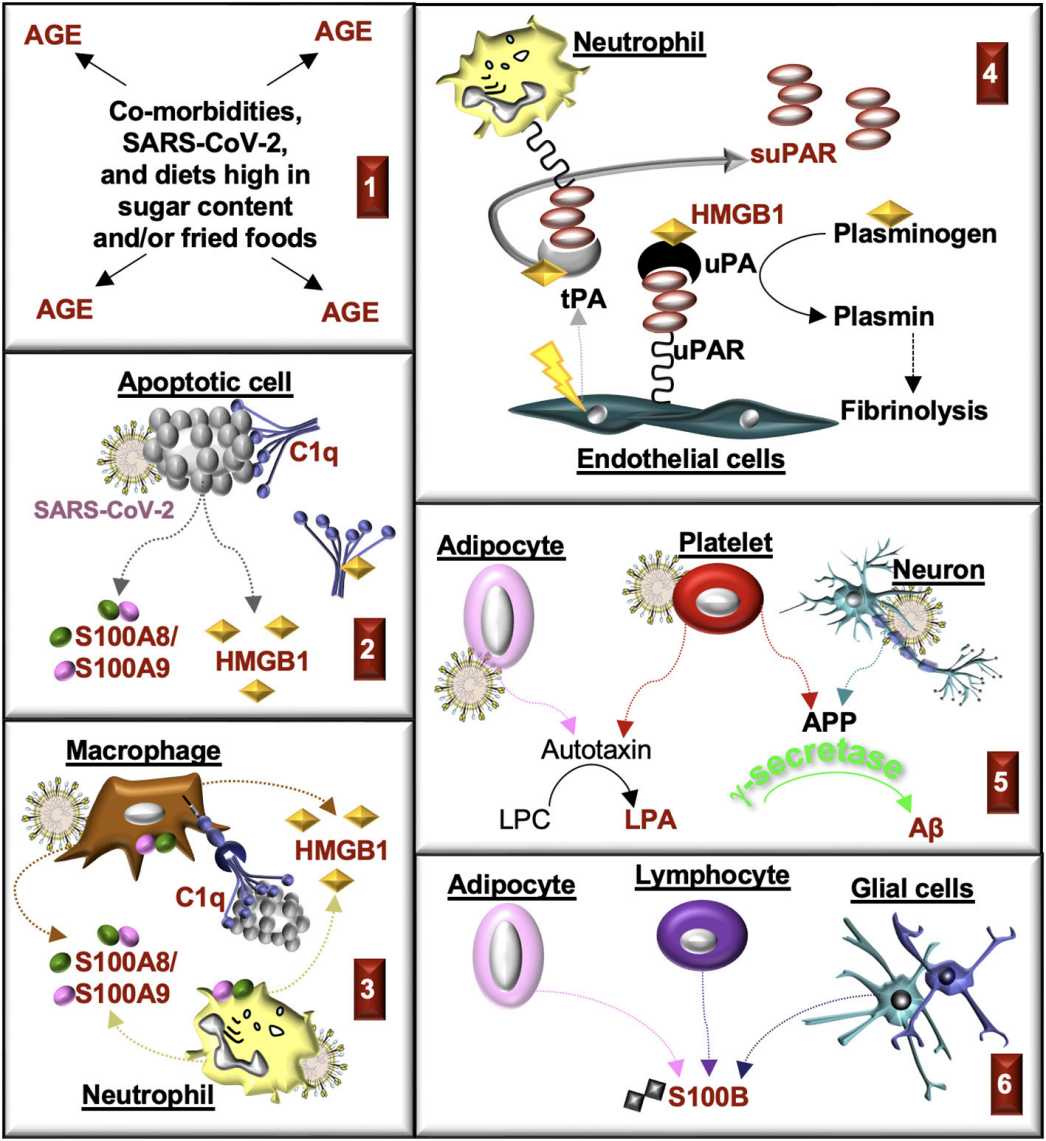
**Production of RAGE ligands during CKD and COVID-19**. RAGE ligands are highlighted in red. **(1)** Advanced glycation end-products (AGE) accumulate in comorbidities associated with CKD and COVID-19. AGE are produced in response to SARS-CoV-2. AGE are also consumed in the diet. **(2)** Cells damaged in response to injury or SARS-CoV-2 infection undergo apoptosis. Apoptotic cells produce S100A8/S100A9 and HMGB1 and generate antigens that bind C1q. HMGB1 can also bind with C1q and form a complex with RAGE and CD305 to induce cell signals that form resolvins and an M2 macrophage phenotype. **(3)** C1q promotes macrophage phagocytosis of apoptotic cells, in part, through binding to RAGE. Myeloid cells release S100A8/S100A9 and HMGB1 in response to injury and infection. **(4)** plasminogen activators (PA; tissue, tPA; urokinase, uPA) are commonly released from endothelial cells in response to injury (tPA) or growth factors (uPA). HMGB1 binds tPA, uPA, and plasminogen, which enhances the rate of plasminogen activation, the formation of plasmin, and fibrinolysis. Urokinase-type plasminogen activator receptor (uPAR) on immune cells, fibroblasts or endothelial cells is cleaved by proteases (e.g., uPA, tPA), which forms soluble uPAR (suPAR). **(5)** Activated platelets and neurons produce amyloid precursor protein (APP), which is cleaved by secretases (β, γ) to form amyloid-β (Aβ) peptides. Aβ is cleared from plasma by the kidney. Activated adipocytes and platelets produce autotoxin, which transforms lysophosphatidylcholine (LPC) into lysophosphatidic acid (LPA). **(6)** S100B accumulates in end-stage kidney disease and severe COVID-19. The source of S100B is not known but might include lymphocytes, glial cells, and/or adipocytes.

SARS-CoV-2 infections promote the production of RAGE ligands in various cell types (Figure 7). Many of these ligands contribute to the pathogenesis of CKD, suggesting that SARS-CoV-2 may initiate or exacerbate kidney injury through RAGE. Here we review the RAGE ligands in CKD and COVID-19.

## Advanced glycation end-products (AGE)

Endogenous circulating AGE are most commonly formed via non-enzymatic glycation of proteins (Figure 2). Glycation of lipids and nucleic acids can also occur via lipid peroxidation. In the diet, exogenous AGE are formed in food and beverages with high sugar content and/or through a browning process (e.g., Maillard reaction, as occurs in heating/frying). Additional receptors [e.g., AGE receptors (AGE-R1–4), scavenger receptors, CD36] that bind AGE may enhance AGE endocytosis and catabolism and inhibit RAGE activation by these molecules. AGE accumulate in tissues with increasing age and contribute to the pathology of diabetes, cancer, obesity, and diseases of the kidney, lung, cardiovascular system, gut, liver, and central nervous system (e.g., Alzheimer, Parkinson) (105, 106). Dysregulation in biological detoxification pathways (e.g., glyoxalase 1, glyoxalase 2, and reduced glutathione) that regulate the formation of the advanced glycation end-product, methylglyoxal, encourage the accumulation of AGE (107).

In a prospective population-based cohort of 643,757 COVID-19 patients, immunodeficiency, hypertension, diabetes, cardiovascular disease, chronic obstructive pulmonary disease (COPD), asthma, kidney disease, cerebrovascular disease, cirrhosis, dementia, severe obesity, and higher age were identified as factors that increased the risk of hospitalization (108). In a meta-analysis of 22,573 COVID-19 patients, cardiovascular, cerebrovascular, and kidney-related comorbidities significantly contribute to greater risk of mortality and increased disease severity (109). Thus, diseases that accumulate AGE in tissues are similarly identified as risk factors in COVID-19 patients.

The N(ε)-Carboxymethyllysine (CML) advanced glycation end-product is an indicator of microvascular inflammation and dysfunction. In comparing autopsied hearts from non-COVID-19 patients and those from the COVID-19 first wave (February–June 2020) and second wave (September–December 2020), CML was found in the endothelium and smooth muscle cells of intramyocardial blood vessels and was elevated in the first wave in association with increased deposits of FVII and FXII clotting factors (110).

Because serum AGE levels are increased in CKD patients and are associated with decreased glomerular filtration rates (9), we hypothesize that AGE may contribute to COVID-19-induced kidney injury.

## Lysophosphatidic acid (LPA)

Extracellular LPA is mainly formed by the removal of the choline moiety from plasma lysophosphatidylcholine by the phospholipase autotaxin, which can be released from adipocytes or secreted by activated platelets (111). LPA binds six LPA receptors (LPA_1−6_), RAGE, and peroxisome proliferator-activated receptor gamma (PPAR-γ) (19). LPA cell signals promote endothelial cell proliferation, smooth muscle cell tissue factor production, and monocyte recruitment, contributing to atherothrombosis (111). This bioactive lipid mediator also functions as a regulatory checkpoint molecule by disrupting T cell engagement with antigen presenting cells, particularly through LPA binding to LPA_5_ (112). In CKD patients, plasma levels of LPA are elevated (113) and in COVID-19 patients, levels of autotoxin are associated with disease severity (114). Whether LPA has a mechanistic role through binding interactions with RAGE or additional receptors in either CKD or COVID-19 remains to be determined.

## Macrophage antigen-1 (Mac-1)

Mac-1, a leukocyte integrin (CD11b/CD18, α_M_β_2_-integrin, CR3), binds soluble ligands (e.g., fibrinogen, factor X, and complement factor iC3b) and cell surface receptors (e.g., CD40L, ICAM-1, platelet GPIbα, and RAGE) to regulate cell signaling, adhesion/extravasation, phagocytosis, thrombosis, and inflammation (115). Heparin also binds Mac-1 and tends to impede the binding of soluble and cell surface ligands to Mac-1 (116). Although the effects of heparin on RAGE binding to Mac-1 have not been fully explored, heparin does antagonize RAGE dimerization (117) and binding to the ligand HMGB1 (118).

Interestingly, Mac-1 and RAGE colocalize on the cell surface of immune cells and Mac-1 binding affinity for ICAM-1 is enhanced by RAGE HMGB1 activation (119). Increased expression of ICAM-1 occurs in the glomeruli, proximal tubular cells, interstitial cells, and endothelial cells in CKD (120). ICAM-1 is also elevated in endothelial cells of COVID-19 postmortem lung biopsies and associated with endothelial dysfunction and pyroptosis (121). The increased production of RAGE ligands in CKD and COVID-19 may therefore activate leukocyte RAGE and enhance Mac-1 binding activity to ICAM-1 on endothelial cells and additional parenchymal cells in the kidney.

Moreover, in a murine model of thrombotic glomerulonephritis, recruitment of neutrophils and platelets into the glomeruli of Mac-1-deficient mice was inhibited and thrombosis and renal failure were prevented, compared to wild-type control mice who manifested glomerular injury (122). In patients with COVID-19 ICU Level 3 ventilatory support, Mac-1 on circulating monocytes and granulocytes is increased compared to healthy and convalescing controls (123). Combined, these research studies support a function of Mac-1 in the recruitment of immune cells, which may include the activity of ICAM-1, RAGE, and RAGE ligands.

## S100 proteins

S100 proteins are a family of small calcium-binding cytoskeletal proteins, forming hetero- or homo-dimers, that are released upon cellular activation or apoptosis. Although RAGE is the most common receptor for many of the 25 different known S100 proteins, additional receptors can bind select S100 proteins and induce inflammatory cell signals (124). Two S100 protein-binding receptors, CD147 (125) and Toll-like receptor (TLR)-4 (126), are also indicated to bind the SARS-CoV-2 spike protein in some studies. RAGE, CD147, and TLR4 all bind the S100 protein, S100A9 (124). Further work is needed to conclusively establish the role of these three cell surface receptors in SARS-CoV2 pathogenesis, and host defense.

The dimer, S100A8/S100A9, is highly expressed in myeloid cells and is an early prognostic marker for AKI in plasma from patients undergoing cardiac surgery (127). Immunohistochemical analysis of biopsy specimens from patients with obstructive hydronephrosis revealed that S100A8/S100A9 proteins are upregulated compared to healthy tissue. Further, in an experimental unilateral ureteral obstruction (UUO) model, S100A9 knockout mice, which are deficient in S100A9 and S100A8, are protected from renal damage and fibrosis compared to wild-type mice (128).

Serum levels of S100A8/S100A9 are also biomarkers of COVID-19 severity and predictors of subsequent ICU admission (129). Blocking S100A9 binding activity with paquinimod in a murine model of lupus inhibited glomeruli complement deposition and reduced hematuria (130). Paquinimod also reduced inflammation and improved survival in murine models of SARS-CoV-2 (131). Whether or not the effects of paquinimod in these studies are due to blocking S100A9 binding to RAGE or additional receptors (e.g., TLR4, CD147) requires further study.

S100B binds RAGE and fibroblast growth factor receptor (FGFR)-1 (124) and is expressed in glial cells, dendritic cells, lymphocytes, and adipocytes (132). Serum S100B is a biomarker for cognitive impairment in patients with ESKD (133) and also an indicator of COVID-19 severity (132). The S100B homodimer is also positively correlated with COVID-19 patient ferritin levels but negatively correlated with lymphocyte counts and percentages (134). Understanding whether lymphocyte apoptosis has a functional role in the increased levels of S100B in ESKD, COVID-19, or RAGE-mediated responses may provide insight into immunity and disease progression.

## Amyloid-beta (Aβ)

Amyloid precursor protein (APP), produced by neurons and platelets, is cleaved by secretases (β, γ) to form Aβ peptides, which bind various lipid, proteoglycan, and neurological receptors, in addition to RAGE. The accumulation of these peptides forms aggregates (fibrils) that may further assemble into plaques (24, 135). The kidney regulates Aβ clearance in the blood, which suggests that kidney dysfunction may play role in the accumulation of Aβ in CKD (136). The SARS-CoV-2 spike protein also binds Aβ (137), which increases infection and reduces Aβ clearance (138). A role for RAGE in this response through Aβ binding interaction (24) or possible downstream γ-secretase production (35) requires further study.

## High-mobility group box-1 (HMGB1)

HMGB1, also known as amphoterin, is a nuclear protein that binds chromosomal DNA, various transcription factors, tissue-type plasminogen activator (tPA), urokinase-type plasminogen (uPA) activator and plasminogen (139). HMGB1 is released by activated macrophages/monocytes and from necrotic or damaged cells. HMGB1 posttranslational modifications, cellular location, redox state, and binding partners regulate binding to various receptors [e.g., TLR2, TLR4, TLR9, CXCR4, RAGE, and T cell immunoglobulin mucin-3 (TIM-3)]. Predominantly through TLR2/4 and RAGE, HMGB1 promotes neutrophil chemotaxis and the production of pro-inflammatory mediators (22).

In CKD, HMGB1 expression in renal tissue and HMBG1 levels in blood and urine are elevated (140). In COVID-19 patients, serum HMGB1 levels increase with disease severity (141). Plasma HMGB1 levels correlate with IL-6 levels and these markers combined predict the mortality of COVID-19 patients in ICU settings (142). Whether HMGB1 has a mechanistic role in disease progression or is simply a marker of disease severity remains to be determined.

HMGB1 activation of RAGE in various human epithelial cell lines promotes mRNA expression of the SARS-CoV-2 receptor, ACE2, and this effect can be blocked by the RAGE inhibitor, FPS-ZM1 (141). The binding of HMGB1 to RAGE can also be blocked by neutralizing HMGB1 with heparin (118) or monoclonal antibodies (143). Further study of these mechanisms and drugs that target RAGE-mediated endocytosis of HMGB1 and its binding partners (e.g., C1q) may offer insight into approaches to regulating inflammatory responses in CKD and COVID-19.

## C1q

The RAGE ligand and complement factor, C1q, recognizes pathogens and apoptotic cells, either directly by binding to antigen-antibody complexes, or indirectly, through associations with pentraxins, such as C reactive protein (CRP). The C1q molecule consists of a collagen-like (cC1q) region that binds the receptor cC1qR and a globular head (gC1q) region that binds gC1qR (93). Another C1q receptor, leukocyte-associated Ig-like receptor-1 (LAIR-1, also known as CD305), forms a complex with RAGE and C1q bound HMGB1, which is also a RAGE ligand. HMGB1 bound to RAGE alone promotes pro-inflammatory cytokine production and an M1 (classical) macrophage phenotype. By contrast, the complex of RAGE:HMGB1:C1q:LAIR-1 induces the production of resolvins (144) and polarizes macrophages to an M2 (alternative) phenotype (29). RAGE alone, or possibly in a complex with the complement receptor, Mac-1/CR3, on the phagocyte surface, also binds C1q and enhances the phagocytosis of C1q opsonized targets (21).

More recently, *in vitro* studies indicate that gC1qR binds to SARS-CoV-2 viral proteins (e.g., spike S1 subunit, membrane-envelope fusion, and nucleocapsid) (145), which may mean the virus competes or synergistically interacts with gC1q for the receptor. In kidney biopsies from COVID-19 patients, C1q deposition is mostly localized to the renal arteries and is rarely detected in glomerular and peritubular capillaries (146). However, in COVID-19 autopsies, increased C1q deposition is also present in glomeruli and tubules, compared to pre-COVID-19 control tissues, perhaps as function of more severe disease (147). It remains to be explored whether RAGE directly binds C1q or forms C1q complexes with SARS-CoV-2, HMGB1, or binds additional receptors/ligands to promote kidney injury in CKD or COVID-19.

## Urokinase-type plasminogen activator receptor (uPAR)

RAGE and the uPAR system are highly integrated. Immune cell Mac-1 forms a lectin domain-dependent membrane complex with uPAR, inducing the expression of a high affinity ICAM-1 binding site (148), which also binds RAGE in *trans* (149). The binding of uPA to the uPAR:Mac-1 complex blocks the Mac-1 high affinity binding site (148), suggesting that uPA may also inhibit RAGE binding to Mac-1. However, in additional studies examining the adhesive and chemotactic functions of Mac-1, HMGB1 stimulated neutrophil recruitment and adhesion in a Mac-1 and RAGE dependent manner, indicating that like uPAR, RAGE partners in *cis* with Mac-1 (119) to enhance Mac-1 adhesive interactions. HMGB1 also binds tPA, uPA and plasminogen, which enhances the rate of plasminogen activation (139). While sRAGE has been shown to inhibit HMGB1-induced Mac-1 adhesion (119), a function of sRAGE in regulating HMGB1-induced activation of plasminogen requires further study.

The cleavage of uPAR on immune cells, fibroblasts and endothelial cells by uPA or additional proteases generates suPAR. Although the functions of suPAR are not fully known, the soluble receptor is indicated to abrogate uPA-mediated plasminogen activation and promote chemotaxis (45). RAGE complexes with the αVβ3-integrin and this complex binds suPAR to promote cell signals (44). Because increased suPAR plasma levels are strong indicators of CKD severity (150), approaches to reduce suPAR protein expression are being considered as therapy for CKD (151). At present, the website clinicaltrials.gov lists several clinical trials that use suPAR plasma levels to guide therapy or assess therapeutic response, but there are, as yet no interventional trials to reduce suPAR plasma or tissue levels. Levels of serum suPAR also increase with COVID-19 severity (152) and is a predictive indicator of in-hospital AKI and the need for dialysis (153). In this regard, targeting RAGE may be an effective approach to reducing the effects of suPAR in these diseases.

## Summary

RAGE ligands (AGE, S100A8/A9, S100B, amyloid-beta, LPA, HMGB1, C1q, suPAR) and sRAGE are elevated in CKD, COVID-19 and many of the co-morbidities that increase the risk of disease severity. AKI and CKD increase the mortality risk in COVID-19 patients. Post-COVID-19 patients with long COVID are at heightened risk for AKI or CKD, and these conditions further increase the mortality risk. Diverse RAGE isoforms, present in plasma and in tissues, and a variety of RAGE ligands contribute to the pathogenesis of these syndromes. RAGE pathway activation is associated with cell injury and dysfunction, in diverse renal cell types, including glomerular cells (podocytes and mesangial cells), tubular epithelial cells, and endothelial cells. RAGE is expressed by infiltrating immune cells, where it promotes immune cell differentiation, recruitment, and activation. RAGE also forms complexes with AT1R, αVβ3, and LAIR-1, which may promote RAAS activity, oxidative stress, and anti-inflammatory responses, respectively. Additional downstream consequences of RAGE activity can include increased TGF-β1 production, epithelial mesenchymal transition, endothelial permeability, and significant systematic inflammation. These effects culminate in glomerulosclerosis, tubulointerstitial fibrosis, cell injury and ultimately organ failure. The accumulation of circulating RAGE ligands coupled with reduced kidney function promotes cognitive impairments and thrombotic complications, increasing disease severity and mortality. Interventions to reduce RAGE and RAGE ligand plasma and tissue levels, as tested in animal models, may offer novel approaches to slow or halt progression of CKD that develops in the absence, presence, or as a consequence of COVID-19.

## Author contributions

CC: methodology, conceptualization, investigation, table formatting, study curation, illustrations, and writing an original draft. JK: methodology, conceptualization, investigation, writing, and editing. All authors contributed to the manuscript and approved the submitted version.

## Funding

This work was supported by the National Institute of Diabetes and Digestive and Kidney Diseases, Intramural Research Program, and the National Institutes of Health Clinical Center.

## Conflict of interest

The authors declare that the research was conducted in the absence of any commercial or financial relationships that could be construed as a potential conflict of interest.

## Publisher's note

All claims expressed in this article are solely those of the authors and do not necessarily represent those of their affiliated organizations, or those of the publisher, the editors and the reviewers. Any product that may be evaluated in this article, or claim that may be made by its manufacturer, is not guaranteed or endorsed by the publisher.
